# Comparing trimodal therapy with radical cystectomy in muscle-invasive bladder cancer: an updated meta-analysis

**DOI:** 10.3389/fsurg.2023.1276746

**Published:** 2023-12-07

**Authors:** Ahmad R. Al-Qudimat, Kalpana Singh, Laxmi K. Ojha, Diala Alhaj Moustafa, Mai Elaarag, Raed M. Al-Zoubi, Omar M. Aboumarzouk

**Affiliations:** ^1^ Surgical Research Section, Department of Surgery, Hamad Medical Corporation, Doha, Qatar; ^2^ Department of Public Health, College of Health Sciences, Qatar University, Doha, Qatar; ^3^ Department of Nursing Research, Hamad Medical Corporation, Doha, Qatar; ^4^ Department of Biomedical Sciences, College of Health Sciences, Qatar University, Doha, Qatar; ^5^ Department of Chemistry, College of Science, Jordan University of Science and Technology, Irbid, Jordan; ^6^ School of Medicine, Dentistry and Nursing, The University of Glasgow, Glasgow, United Kingdom

**Keywords:** cancer, bladder preserving, radical cystectomy, muscle-invasive, trimodal

## Abstract

**Background:**

We conducted this meta-analysis to compare the two muscle-invasive bladder cancer (MIBC) treatment modalities in terms of cancer-specific survival (CSS) and other outcome indicators.

**Method:**

A systematic review and meta-analysis were performed in accordance with the Preferred Reporting Items for Systematic Reviews (PRISMA) guidelines. The search was conducted using various academic databases including Scopus, PubMed, Cochrane database, EMBASE, Chinese biomedical literature database, Wan fang databases, and China National Knowledge Internet databases between 1966 and December 2023. This review protocol was registered in the International Prospective Register of Systematic Reviews (PROSPERO) No. (*CRD42023398977*).

**Result:**

This study included a total of 54,816 patients diagnosed with bladder cancer from 14 studies, of which 6,228 patients were assigned to the trimodal therapy (TMT) group and 48,588 patients were assigned to the radical cystectomy (RC) group. Based on the results, the RC group exhibited a higher rate of survival than the TMT group [pooled hazard ratio (HR) = 1.23, 95% CI: 1.18–1.28, *Z* = 1.46, *P* < 0.001]. In terms of CSS, patients in the RC group had a longer CSS compared with those in the TMT group (pooled HR = 1.47, 95% CI: 1.29–1.67, *Z* = 5.893, *P* < 0.001). Compared with RC, TMT is significantly associated with an increased risk of both types of mortality (pooled HR: 1.30, *P* < 0.001).

**Conclusion:**

Overall, the findings of this meta-analysis suggest that RC treatment may be associated with improved overall survival. Moreover, it was observed that cancer-specific survival was significantly prolonged among patients in the RC group as opposed to those who received TMT. In addition, it was shown that patients who received TMT exhibited a higher risk of all-cause mortality when compared with those who underwent RC.

## Introduction

The incidence of bladder cancer, commonly referred to as urothelial or urinary bladder cancer, is progressively increasing worldwide, particularly in developed nations. Presently, it is ranked 10th among the most widespread types of cancer globally (1, 2). Bladder urothelial carcinoma has a reasonably high incidence rate, particularly in developed countries, ranking as the seventh most common tumor in males and the 11th in both sexes (3). In terms of both cost and fatality, bladder cancer stands out as the most burdensome among urologic malignancies. In 2019 alone, approximately 80,470 new cases were diagnosed, leading to an estimated 17,670 deaths caused by this disease (4). The 5-year survival rates for individuals diagnosed with bladder cancer were found to be 34% for those with localized disease, 7% for those with regional disease, and 5% for those with metastatic disease. The survival rates of those diagnosed with progressed and metastatic bladder cancer were found to be much lower (5).

At the time of the diagnosis, approximately 20%–30% of the lesions will have invaded the muscle; this necessitates a radical cystectomy (RC) in conjunction with pelvic lymphadenectomy, which is considered the “gold standard” treatment (6, 7). Within 30 days following the surgery, the complication rates have been documented to reach as high as 58%–77%, and approximately 27% of patients require readmission. Partial cystectomy (PC) combined with bilateral pelvic lymph node dissection has been offered as an alternative treatment for these conditions, following a thorough process of patient screening. Based on the available data, between 5% and 10% of patients meet the selection requirements for undergoing a PC procedure (8). In the 1950s, partial cystectomy for muscle-invasive urothelial carcinoma was widely employed, but this approach has since fallen out of favor due to unacceptable rates of recurring bladder cancer (40%–78%), which can largely be attributable to the insufficiently stringent selection criteria employed (9–11).

Patients with clinically staged MIBC and treated with contemporary methods that preserve the bladder have a chance of achieving complete response (CR) rates ranging from 60% to 80%, the 5-year disease-specific survival (DSS) rates fall within the range of 60%–70%, while the rates of survival with the bladder intact range from 40% to 45% (12, 13).

We conducted this meta-analysis to compare the two MIBC treatment modalities in terms of CSS and other outcome indicators because we think that cumulative evidence from trials should be more trustworthy.

## Method

We performed this review based on *a priori*-defined protocol and according to PRISMA and meta-analysis guidelines (14). The review protocol has been registered in the International Prospective Register of Systematic Reviews (PROSPERO) under the registration number (*CRD42023398977*).

### Search strategy

We searched Scopus, PubMed, Cochrane database, EMBASE, Chinese biomedical literature database, Wan fang databases, and China National Knowledge Internet databases between 1966 and December 2023. We used the terms bladder cancer, trimodality, chemotherapy, radiotherapy, and cystectomy. The phrases used for the Medical Subject Heading (MeSH) search included: [“bladder cancer” (MeSH)] or “bladder preservation” (MeSH) or “bladder-sparing” (MeSH) and “trimodality treatment” (MeSH) or “TMT” (MeSH) and “chemotherapy” (MeSH) and “chemoradiation” (MeSH) and “radiotherapy” (MeSH) and “chemoradiotherapy” (MeSH) and “cystectomy” (MeSH). We considered including all original papers and retrieved all available records.

### Study eligibility

We defined suitability of this review be utilizing the PICO question (P = Patient population, I = Intervention, C = Comparator, and O = Outcomes). The studies that were included in the analysis were selected based on the following criteria: (1) adult (2) studies have comparting between RC and TMT, (3) compared outcomes between RC and TMT with patients with muscle-invasive bladder cancer (MIBC), and (4) local or local advance tumor. The exclusion criteria were as follows: (1) duplicate reports (including identical patient information), (2) insufficient data, and (3) reviews and other reports.

### Search strategy and study selection

According to the inclusion criteria, two authors (AA and LO) screened and evaluated the relevant studies. Where opinions differed, discussions were held with the lead author (OA) until an agreement was reached.

### Data extraction

We extracted data independently by authors using a standard excel sheet. The information extracted from the studies included studies characteristics (author names, publication years, study design, total study population, country, timeframe of study), outcome [cancer-specific survival (CSS) and Charlson comorbidity score (CCS) after TMT or RC treatment], and mortality.

### Heterogeneity assessment

We used the *I*^2^ statistic and a visual evaluation of the forest plots to assess the presence of heterogeneity. Specifically, *I*^2^ values of 50% were utilized to indicate low and high levels of heterogeneity, respectively (15).

### Publication bias

We conducted a regression test to assess the presence of funnel plot asymmetry across all potential outcomes.

### Statistical analysis

Variance and log hazard ratio (HR) has been implemented as the summary outcome measures in all studies in this meta-analysis. We calculated the HR at the 95% confidence interval (CI) for mortality, cancer-specific survival, and overall survival (OS). Across all the included papers, the risk ratio (RR) was adopted as the summary outcome measure and was calculated with a 95% CI of the data for each study to compare TMT with RC. Also, the risk ratio with 95% CI was applied to compare the clinical T stage, cancer grade, Eastern Cooperative Oncology Group (ECOG) score, and CCS of patients with TMT or RC. The *Z*-test was used to determine the statistical significance of the summary RRs. The *I*^2^ test and chi-square test were applied to assess the level of heterogeneity among the studies. To determine the level of heterogeneity, the *I*^2^ value was employed (*I*^2^ = 25%, no heterogeneity; *I*^2^ = 25%–50%, moderate heterogeneity; I2% > 50%, high or extreme heterogeneity). If *P* < 0.05, a statistically significant heterogeneity in the statistics was considered to exist. The random-effects models (DerSimonian–Laird method) were used to assess the pooled RR and to test the reliability of the results. For evaluating publication bias, funnel plots with the Begg's rank test were employed (16). STATA 17.0 was used for all the analysis. *P*-values that were <0.05 were considered statistically significant. All statistical tests were two-sided.

## Results

**Figure 1:**
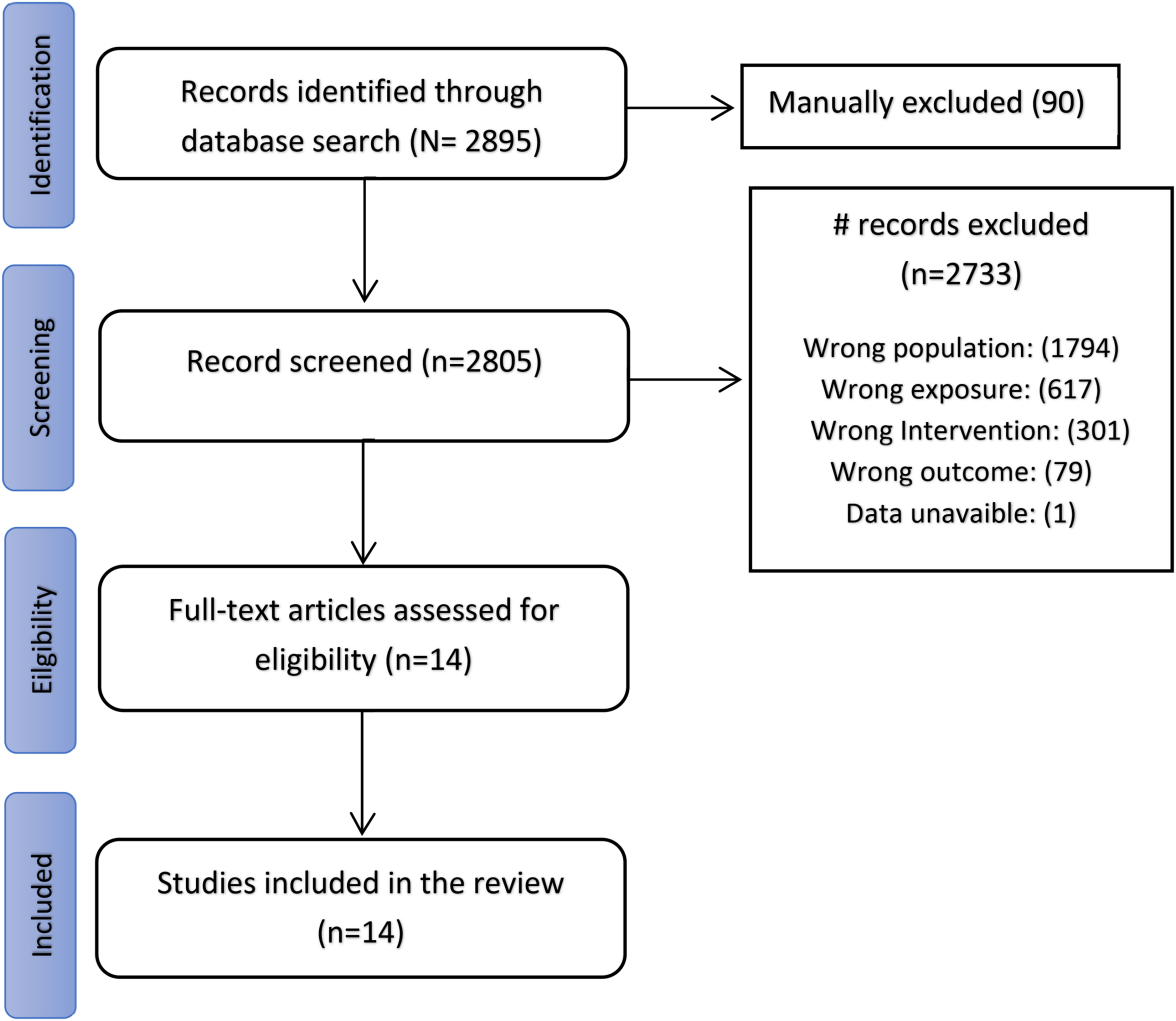
PRISMA diagram of literature search.

The initial search included 2,895 studies that were potentially relevant to the topic under investigation. Following the initial screening process, 90 studies were excluded due to duplication, and 2,732 studies were further excluded based on the evaluation of their titles or abstracts, or failure to meet the eligibility criteria. A total of 13 studies were deemed relevant and therefore included in the review (Figure 1).

### Risk of bias assessment

**Figure 2:**
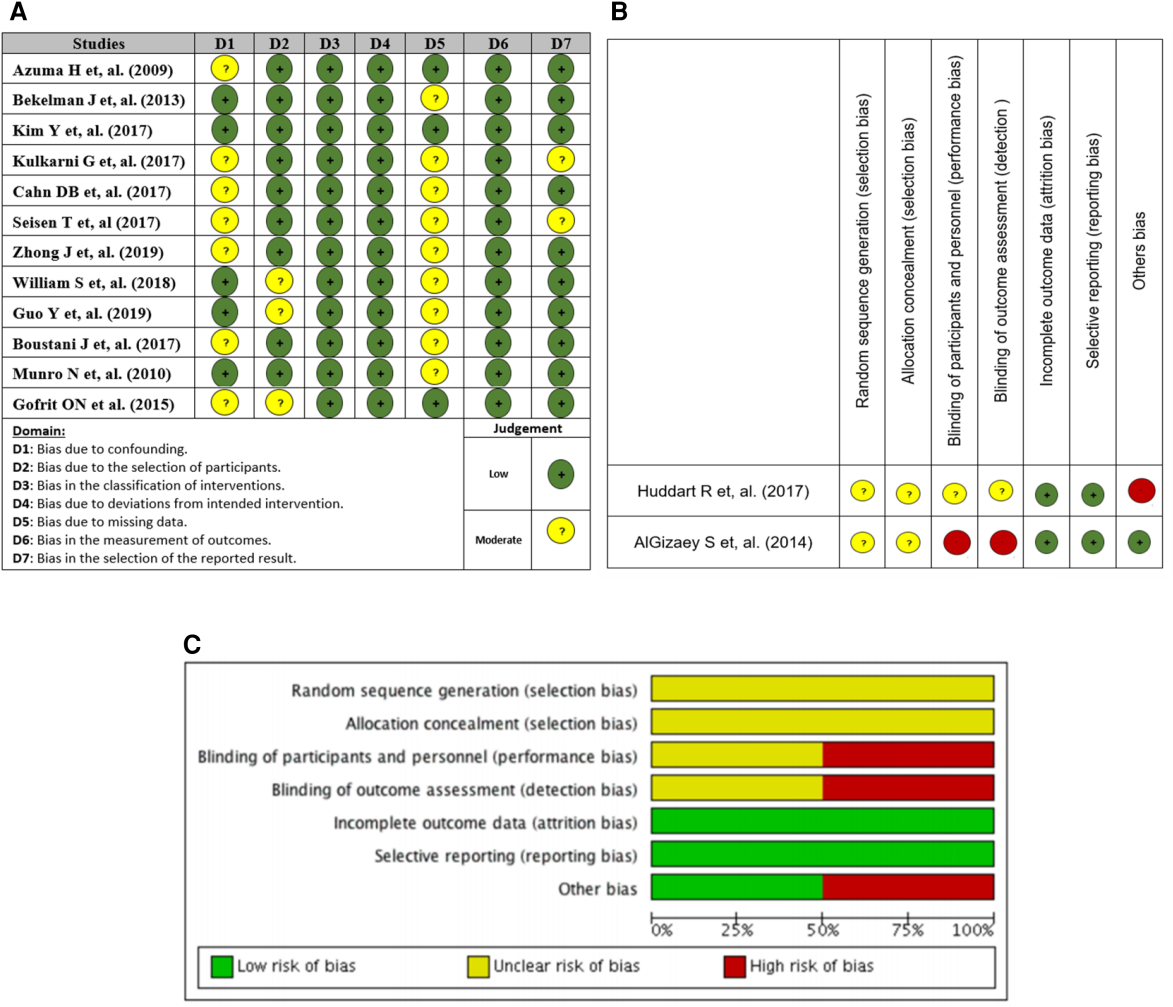
(**A**) Traffic light plot of risk of bias assessment (non-randomized studies), (**B**) Cochrane tool, and (**C**) risk of bias across studies Cochrane tool.

A total of 12 non-randomized intervention studies (5, 17–27) were evaluated using the traffic light diagram to assess the risk of bias. The risk of bias was low across all studies in relation to the classification of the intervention, deviations from the intended intervention, and measurement of outcomes. A total of nine studies were evaluated to have a moderate risk of bias due to missing data, while seven studies were found to have a moderate risk of bias due to confounding factors, and three studies were identified as having a moderate risk of bias due to participant selection (Figure 2).

The two randomized clinical trials (28, 29) were evaluated using the Cochrane Bias Risk Tool. The study revealed a low likelihood of bias resulting from exclusion and reporting bias, whereas the risk of selection bias in the two components remains unclear. The assessment of one study indicated an unclear risk of bias, while another study was deemed to have a high risk of bias in relation to performance and detection bias (Figure 2).

### Study characteristics

A total of 14 studies from eight different countries were included, namely one from China, one from Egypt, one from France, one from Korea, one from Israel, one from Takatsuki, three from Great Britain, and five from the United States. The meta-analysis comprised a total of 54,816 patients diagnosed with bladder cancer. Among these, 6,228 patients were from the TMT group, while the remaining 48,588 patients were from the RC group. Ten studies were retrospective, one study was a prospective cohort study, and three studies were randomized controlled trials (RCTs). CSS was reported in five studies, with two of these studies reported on mortality outcomes. Table 1 provides an overview of all the details of the research characteristics. Most of the patients included in the study exhibit urothelial carcinoma as their pathology grade, while others present with a different form of carcinoma. Four studies provided the ECOG score, while 12 studies assessed the clinical T stage.

**Table 1:** Characteristics of the included studies.

Author (years)	Country	Study design	Arm	Sample size	Male vs. female	Age (mean)	T stage (<2 vs. ≥2)	Cancer grade (UC vs. others)	ECOG Score 0 vs. ≥1 (%)	CCS (%) 0 vs. 1 vs. ≥2	Chemo radiotherapy
Azuma et al. (5)	Takatsuki	Prospective	RC	62	48 vs. 14	64	50 vs. 50	56 vs. 6	46.8 vs. 53.2	Ns	Cisplatin:100–300 mg
			TMT	62	43 vs. 19	72	24.2 vs. 75.8	55 vs. 7	33.9 vs. 66.1		Radiation: 60.4 Gy
Bekelman et al. (17)	USA	Retrospective	RC	1,426	892 vs. 534	75.4	Ns	Ns	Ns	Ns	Ns
			TMT	417	300 vs. 117	79.3					
Kim et al. (22)	Korea	Retrospective	RC	308	260 vs. 48	65	47.1 vs. 52.9	308 vs. 0	40 vs. 16	Ns	Gemcitabine: 1,000 mg/m^2^ and Cisplatin:70 mg/m^2^
			TMT	32	25 vs. 7	77	56.3 vs. 43.8	32 vs. 0	40 vs. 16		Radiation: 46 Gy
Kulkarni et al. (23)	UK	Retrospective	RC	56	41 vs. 15	71	73.2 vs. 26.8	Ns	Ns	Ns	Cisplatin: 40 mg/m^2^
			TMT	56	40 vs. 16		67.9 vs. 32.1				Radiation: 66 Gy
Cahn et al. (19)	USA	Retrospective	RC	22,680	17,055 vs. 5,625	80.1	54.2 vs. 45.8	20,503 vs. 2,177	Ns	Ns	Any chemotherapy
			TMT	1,489	1,112 vs. 377	94.2	81.9 vs. 18.1	1,330 vs. 159			Radiation:50–80 Gy
Seisen et al. (25)	USA	Retrospective	RC	11,586	8,725 vs. 2,861	68.1	80.1 vs. 19.9	Ns	Ns	70.3 vs. 23 vs. 6.7	Any chemotherapy
			TMT	1,257	955 vs. 302	74.8	82.1 vs. 17.9			68.5 vs. 23.3 vs. 8.2	Radiation: 60–65 Gy
Zhong et al. (27)	USA	Retrospective	RC	7,276	5,499 vs. 1,777	67.39	86.5 vs. 13.52	7,276 vs. 0	Ns	69.46 vs. 23.67 vs. 6.87	Any chemotherapy
			TMT	1,178	863 vs. 315	75.21	88.71 vs. 11.29	1,178 vs. 0		65.62 vs. 24.96 vs. 9.42	Radiation:64.8 Gy
William et al. (26)	USA	Retrospective	RC	2,448	1,516 vs. 932	75.8	39.5 vs. 60.5	2,387 vs. 61	Ns	56.6 vs. 26.4 vs. 17	Cisplatin or fluorouracil and mitomycin C
			TMT	752	532 vs. 220		70.7 vs. 29.3	709 vs. 43		47.1 vs. 27.4 vs. 25.5	Radiation:60–66 Gy
Guo et al. (21)	China	Retrospective	RC	2,420	1,611 vs. 809	65	21.6 vs. 78.4	0 vs. 25	Ns	Ns	Ns
			TMT	478	359 vs. 119	67.5	63.2 vs. 36.8	0 vs. 20			
Huddart et al. (29)	UK	RCT	RC	25	22 vs. 3	67.6	22 vs. 1	Ns	Ns	0 vs. 52 vs. 40	Any chemotherapy
			TMT	20	18 vs. 2	63.3	14 vs. 4			0 vs. 56 vs. 16	Radiation:60–66 Gy
Boustani et al. (18)	France	Retrospective	RC	92	68 vs. 24	82.82	60 vs. 84	Ns	40 vs. 16	0 vs. 0 vs. 56	Ns
			TMT	72	40 vs. 32	83.64	57 vs. 87		40 vs. 16	0 vs. 0 vs. 56	
Munro et al. (24)	UK	Retrospective	RC	96	64 vs. 32	71	47 vs. 16	Ns	Ns	Ns	Ns
			TMT	302	196 vs. 106	66	167 vs. 135				
Gofrit et al. (20)	Israel	Case control	RC TMT	33 33	25 vs. 8 26 vs. 7	72.8 73.6	Ns	Ns	Ns	Ns	Ns
Algizaey et al. (28)	Egypt	RCT	RC	80	62 vs. 18	55.6	61 vs. 19	0 vs. 79	0 vs. 80	Ns	Ns
			TMT	80	65 vs. 15	58.6	60 vs. 20	0 vs. 7+	0 vs. 80		

TMT, trimodal therapy; RC, radical cystectomy; RCT, randomized controlled trials; Ns, not stated.

**Figure 3:**
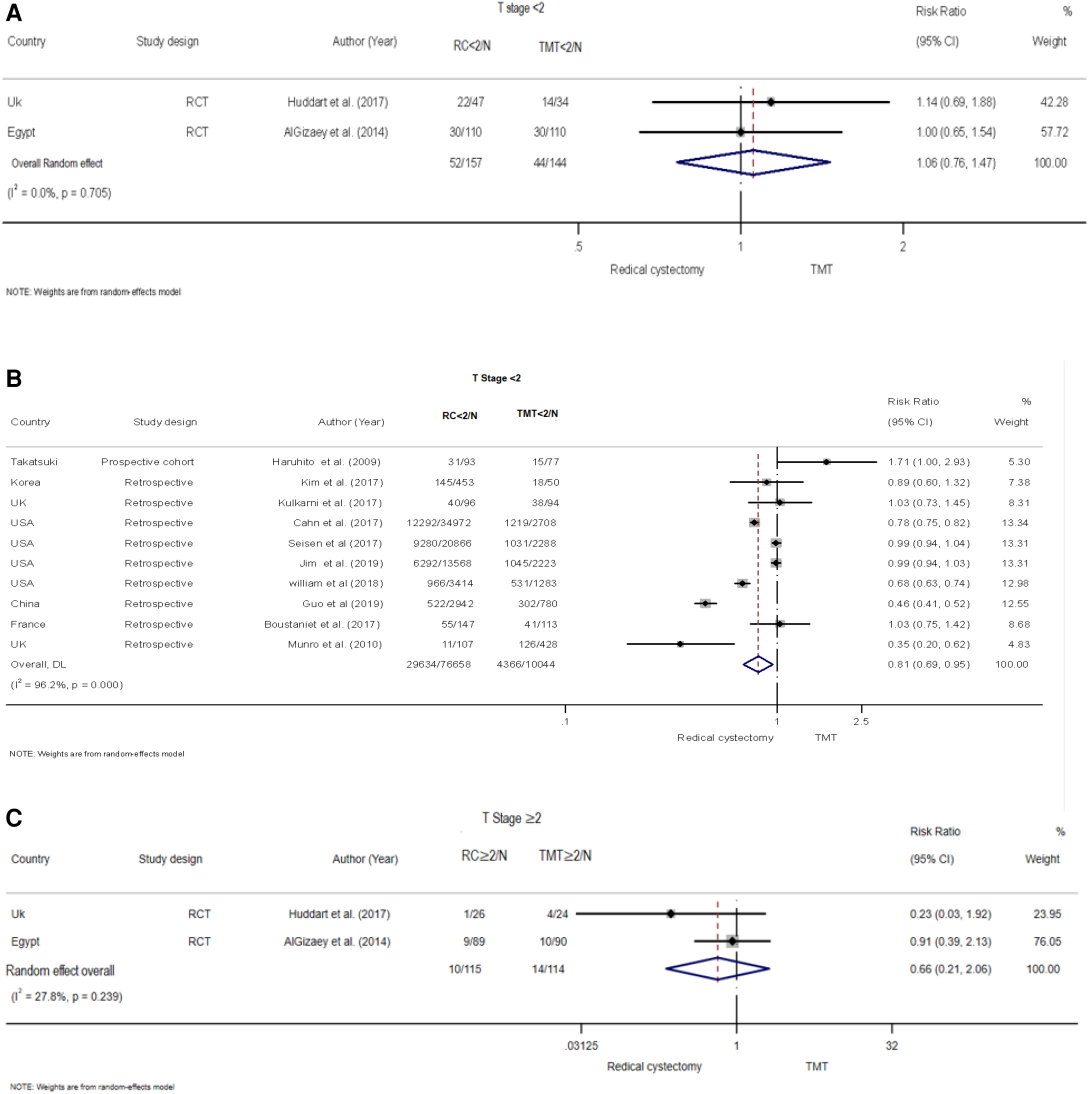

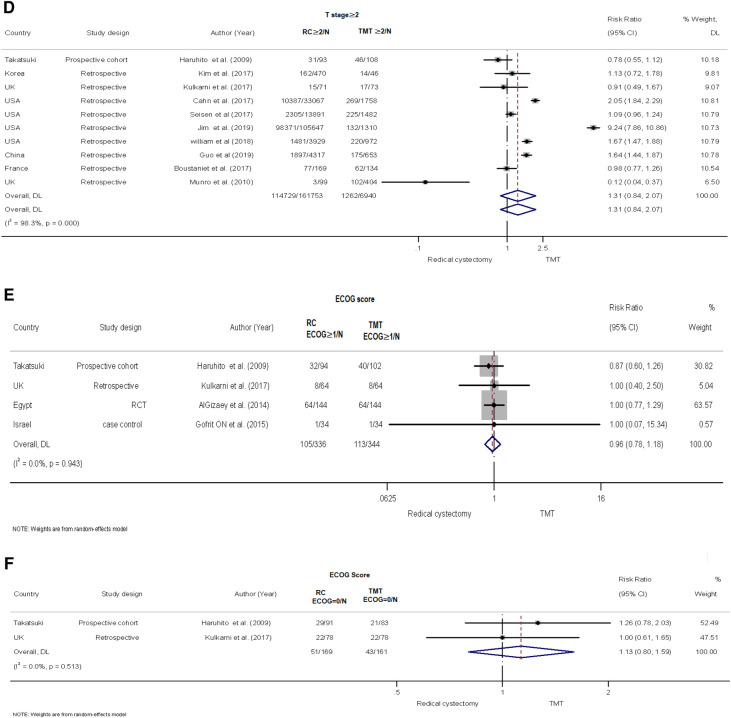
Forest plot comparing clinical T stage and eastern cooperative oncology group (ECOG) in patients receiving trimodal therapy (TMT) vs. radical cystectomy (RC). (**A**) T stage <2 for RCT, (**B**) T stage <2 for retrospective studies, (**C**) T stage ≥2 for RCT, (**D**) T stage ≥2 for retrospective studies, (**E**) ECOG ≥1, and (**F**) ECOG = 0.

The risk of clinical T stage <2 group between TMT and RC was examined in 10 retrospective and one prospective study involving 54,545 participants (5, 18, 19, 21–27). The random-effects model was employed to calculate the pooled risk due to the presence of evident heterogeneity among these studies (*I*^2^ = 95.8%, *Q* = 239.1, *P* < 0.001). The data illustrated that the risk of the RC group was lower than that of the TMT group (pooled RR = 0.81, 95% CI: 0.69–0.95, *Z* = −2.587, *P* < 0.010, (Figure 3B).

Two RCTs with 301 patients (28, 29) compared the risk of clinical T stage <2 group between TMT and RC (Figure 3A). Using a random-effects model, the pooled risk was higher in the RC group than in the TMT group, but the difference was not statistically significant (pooled RR = 1.06, 95% CI: 0.76–1.47, *Z* = 0.324, *P* = 0.746). No heterogeneity was found between the two RCTs (*I*^2^ = 0%, *Q* = 0.14, *P* = 0.705).

There was no statistical significance in the difference between TMT and RC for clinical T Stage ≥2 group for retrospective and prospective studies demonstrated in the pooled RR results (5, 18, 19, 21–27) (pooled RR = 1.31, 95% CI: 0.84–2.07, *Z* = 1.18, *P* = 0.237) (Figure 3D). In the RCT (28, 29), the pooled risk ratio was found to be 0.66, with 95% CI: 0.21–2.06, *Z* = −0.722, *P* = 0.470 (Figure 3C). These findings indicate that there was no statistical difference observed between TMT and RC in terms of ECOG score for both the 0 and ≥1 groups (5, 20, 23, 28) (Figures 3E,F).

### Cancer grade

**Figure 4:**
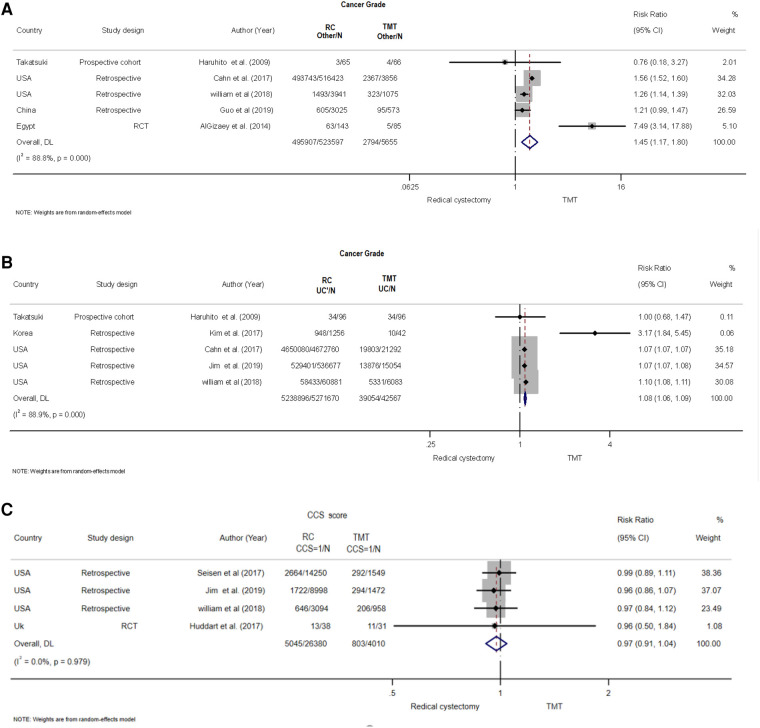

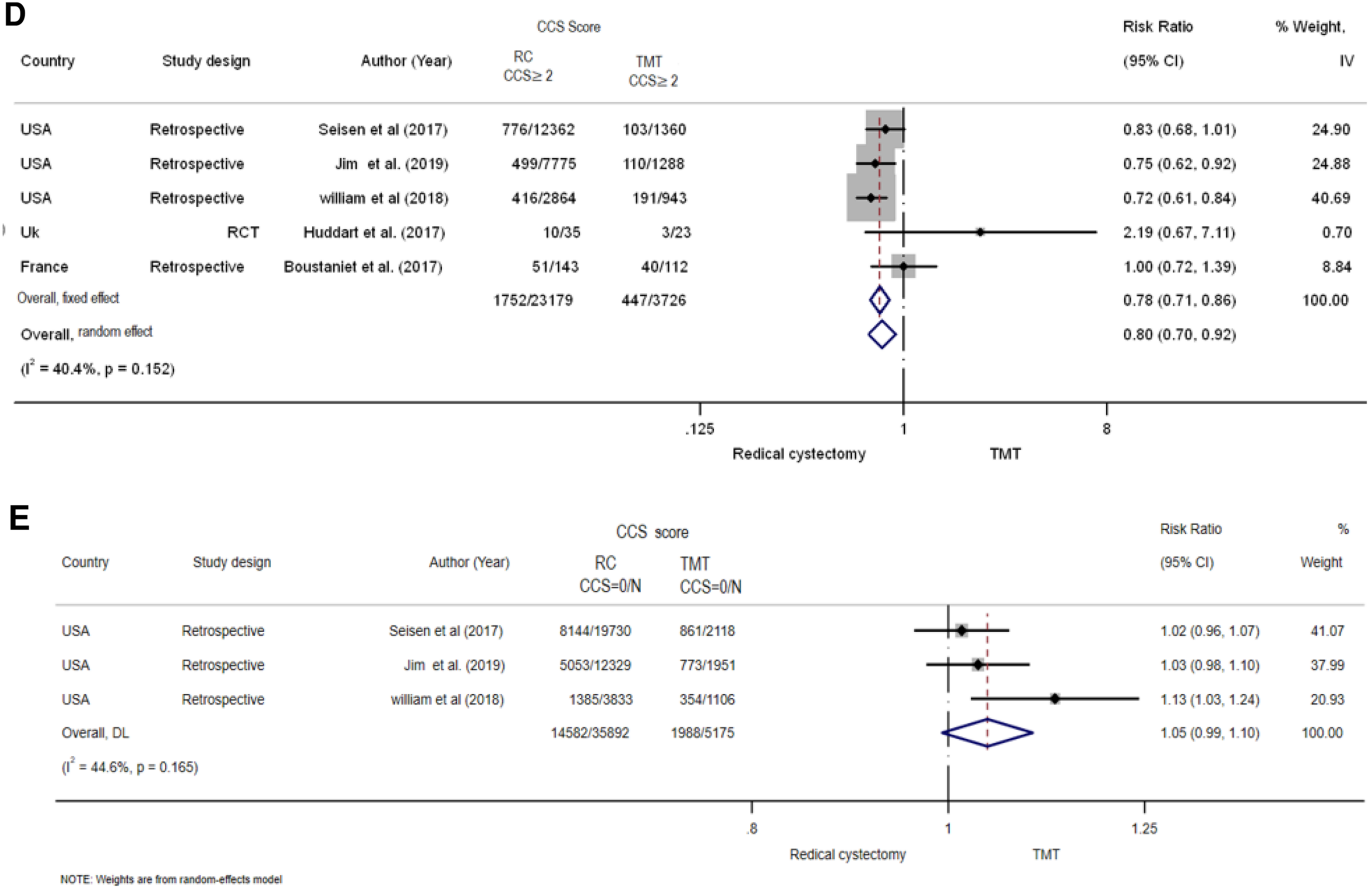
Forest plot comparing cancer grade and Charlson comorbidity score (CCS) in patients receiving trimodal therapy (TMT) vs. radical cystectomy (RC). (**A**) Cancer grade others, (**B**) cancer grade for UC, (**C**) CCS ≥2, (**D**) CCS = 0, and (**E**) CCS = 1.

Five retrospective studies (5, 18, 19, 21–27) with 36,287 patients examined the cancer risk for urothelial diseases between TMT and RC. The studies exhibited apparent heterogeneity (*I*^2^ = 88.9%, *Q* = 36.2, *P* < 0.001), and the pooled risk was calculated using the random-effects model. The results showed that the risk of the RC group was higher than that of the TMT group (pooled RR = 1.08, 95% CI: 1.061.09, *Z* = 11.575, *P* < 0.001) (Figure 4B).

A total of 30,551 patients were included in five studies, three of which being retrospective, one being prospective, and one being an RCT (5, 19, 21, 26, 28), to assess the risk of cancer grade for various types of cancer between TMT and RC (Figure 4A). Using a random-effects model, the pooled risk was found to be significantly higher in the RC group compared with the TMT group (pooled RR = 1.45, 95% CI: 1.17–1.47, *Z* = 3.437, *P* = 001. There was clear heterogeneity between the studies (*I*^2^ = 88.8%, *Q* = 35.67, *P* < 0.001).

### Charlson comorbidity score

To determine the pooled risk of CCS score for ≥2 between TMT and RC (Figure 4C), a total of 24,706 patients were included from five studies, four of which were retrospective studies and one was an RCT (18, 25–27, 29). A random-effects model revealed that the pooled risk was significantly lower in the RC group than that in the TMT group (pooled RR = 0.80, 95% CI: 0.70–0.92, *Z* = −3.066, *P* = 002. The studies exhibited a moderate amount of heterogeneity (*I*^2^ = 40.4%, *Q* = 6.7, *P* = 0.152).

There was no significant difference found in the pooled risk of CCS score for 0 as well as 1 group between RC and TMT group (Figures 4D,E) (25–27, 29).

## Overall survival

Based on the information provided, it appears that a comparative analysis is being conducted between two treatment methods, namely TMT and RC. The comparison is based on the overall survival outcomes from 11 studies.

The presence of heterogeneity among the studies is evident, indicated by an *I*^2^ value of 70.2% and a *P*-value of <0.001. The random-effects model was employed to calculate the pooled hazard ratio to account for this heterogeneity. The data showed that the OS of the RC group was higher than that of the TMT group [the pooled HR 1.23 (95% CI: 1.18–1.28), *Z* = 10.7, *P* < 0.001] (Figure 3A) (5, 17–20, 22, 25–29). This result suggests that the RC treatment may be associated with better overall survival compared with the TMT treatment.

## Cancer-specific survival

**Figure 5:**
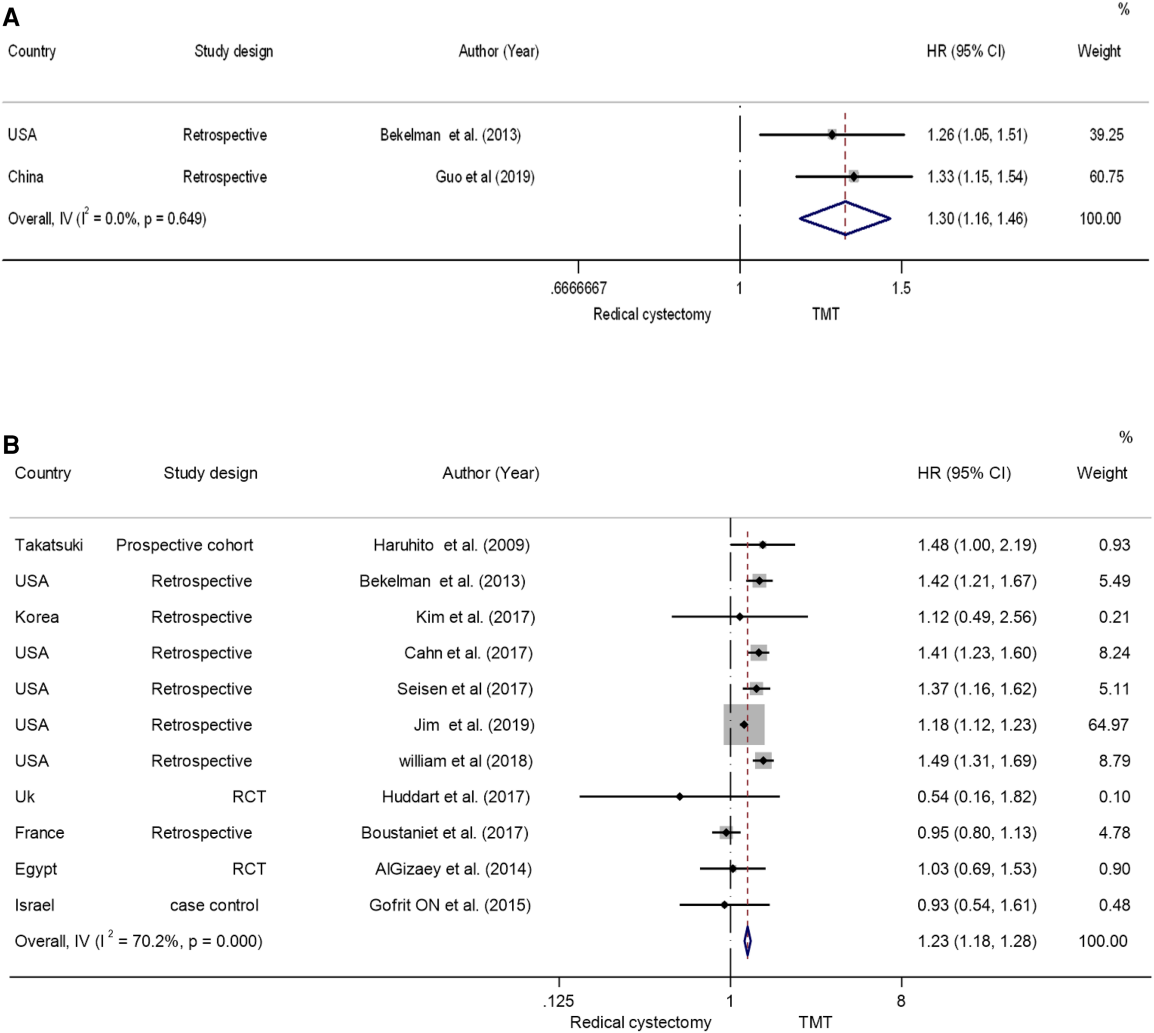
Forest plot comparing (**A**) cancer-specific survival (**B**) overall survival in RCTs in patients receiving trimodal therapy (TMT) vs. radical cystectomy (RC).

Six studies reported CSS in relation to both TMT and RC (17, 20, 22, 25, 26, 28). The random-effects model was chosen to assess the combined HR in order to account for the absence of significant heterogeneity between studies (*I*^2^ = 0%, *P* = 0.58). The results showed that the patients in the RC group had a longer CSS compared with the patients in the TMT group (pooled HR = 1.47, 95% CI: 1.291.67, *Z* = 5.893, *P* < 0.001) (Figure 5A).

## Mortality

Two studies (17, 21) reported the all-cause mortality and bladder-specific cancer mortality between TMT and RC; the pooled hazard ratio findings suggest that TMT is linked with a substantial increase in both types of mortality when evaluated to RC. The pooled HR was found to be 1.30, 95% CI: 1.16–1.46, *Z* = 4.546, *P* < 0.001 (Figure 5B). This suggests that patients receiving TMT have a higher risk of all-cause mortality compared with those receiving RC.

## Publication bias

The present meta-analysis of 14 studies did not identify any significant impact of small study size. The publication bias was calculated using Begg's rank test, yielding a *P*-value of 0.099, which did not reach statistically significance. Evidence was found to support the absence of publication bias.

## Discussion

Muscle-invasive bladder cancer has historically been treated with radical cystectomy; however, trimodal therapy (TMT) has become a viable therapeutic alternative for some patients (30). Due to the lack of top-tier high-quality comparative studies between the two alternatives, we sought to compare the clinical outcomes between these two different treatment modalities by analyzing the best current literature available. The outcomes of interest in this study were clinical T stage, cancer grade, Charlson comorbidity score, overall survival, cancer-specific survival, and mortality. In this study, we find that among MIBC patients, those who underwent RC exhibited better OS, cancer-specific survival, and lower mortality compared with patients who underwent TMT. In addition, patients with a clinical T stage <2 were susceptible to TMT therapy. There was no observed difference between TMT and RC in cases when the clinical T stage was >2. In terms of CSS, it was shown that patients who had a CSS score of ≥2 had a higher likelihood of undergoing TMT. No significant difference was observed between TMTM and RC in patients with a CSS score of <2.

RC was considered the gold standard for managing invasive bladder cancer. However, modern therapeutic options lean toward organ preservation, optimizing the quality of life while ensuring treatment efficacy (31). The Radiation Therapy Oncology Group (RTOG) has conducted six clinical trials that investigated bladder-preservative alternatives. A total of 415 patients were enrolled in these trials with a survival rate of 50%. These alternatives were aimed to increase the tolerability to chemotherapy, thus improving compliance to the treatment and ultimately resulting in improved overall survival rates. However, these were not aimed to replace RC, but rather to provide an alternative for those who cannot undergo surgery (31).

When comparing TMT and RC, conflicting evidence exists. One meta-analysis demonstrated the superiority of TMT over RC in improving the 5-year OS rate (32). In contrast to an older meta-analysis that demonstrated no difference between the two interventions, it did indicate an additional benefit associated with RC regarding cancer-specific survival (33). Moreover, another meta-analysis illustrated neutrality between the two interventions regarding all survival outcomes (34). A recent meta-analyses published in 2020 showcased that TMT and RC are comparable in <10-year OS. However, in the overall survival period of more than 10 years, RC was superior to TMT. It can be concluded that TMT might be a viable treatment option for a selected group of patients (35).

In addition to the efficacy outcomes, it is crucial to examine the safety outcomes related with RC, which have been associated with sexual dysfunction and the need for urinary diversion that required external drainage devices. These might affect the mental and emotional status of the patients. TMT overcomes these side effects by sparing the bladder function, and it has been associated with an improved quality of life (QOL) compared with RC as patients have better body perception (36). Another two cross-sectional studies showed that TMT is associated with enhanced QOL (37, 38). On the other hand, TMT is associated with hematological, gastrointestinal (GI), and urogenital (GU) side effects. However, most studies have concluded that TMT is tolerable and has a well-established safety profile, as the results showed that there is a slight increase in grade 3 or 4 acute adverse events (AEs) with TMT, and that these events were predominantly gastrointestinal toxic effects (35–39).

With limited resources setting, the cost-effectiveness of treatment can be as important as clinical outcomes. TMT is associated with a higher cost than RC with a median difference of $127,815 at 2 years, in addition to the outpatient costs including radiology, medications, pathology/laboratory, and other professional services, while RC exhibited higher inpatient costs (40). In certain scenarios, TMT can be a cost-effective option such as in patients aged 65 and older (41), or in academic centers (academic hospitals), while RC is more cost-effective on the population level (42).

## Limitations

Some inherent limitations in this study should be acknowledged and put into consideration when interpreting the results. Firstly, the primary sources of the data mainly consisted of retrospective studies, which can be prone to certain limitations such as low quality of documentation, missing data, and selection bias. In addition, using retrospective studies in the analysis might explain the observed heterogeneity in the outcomes, as the data were not originally collected for the purpose of conducting a comparative analysis, resulting in a variation of treatment protocols and the presence of uncontrolled confounding factors, such as using neoadjuvant therapy, variations in radiation dose and type, and the choice of chemotherapy agent. Second, our systematic review concerned the proportion of retrospective studies and the small sample size of the RCTs included. This hampered our ability to discern differences and increased the heterogeneity of the estimates for each outcome. Moreover, we found selection biases in patients receiving different therapies, including variations in performance status, tumor and nodal status, and treatment management (e.g., different radiation and chemotherapy interventions) between institutions. Variant histologies are another limitation. The influence of these variant histologies, as defined by the novel World Health Organization classification, on interventions is not well understood. These variant histologies can provide different prognostic and diagnostic results, which may lead to different therapeutics for each variant type (43–45). This selection bias has the potential to further increase heterogeneity. Despite the limitations, this study serves as a basis for clinicians involved in treating MIBC, as it provides a summary of the existing literature comparing these two alternative treatment options.

## Conclusion

This study provides evidence of a positive clinical association between RC and TMT. The findings indicate that patients with MIBC who are eligible for treatment had a greater likelihood of prolonged overall survival, cancer-specific survival, and mortality survival while undergoing RC compared with TMT. Our findings may be used to support clinicians in managing MIBC patients. Future work should also extend the scope of investigation and assess clinical outcomes in a younger population with longer expected life spans, in addition to measuring economic outcomes in different regional settings.

### Key points

• The updated meta-analysis of the comparison between trimodal therapy (TMT) to radical cystectomy (RC) in muscle-invasive bladder cancer.
• Comparing cancer grade and Charlson comorbidity score (CCS) in patients receiving trimodal therapy vs. radical cystectomy. (A) Cancer grade others, (B) Cancer grade for UC, (C) CCS ≥2, (D) CCS = 0, and (E) CCS = 1 in patients receiving trimodal therapy vs. radical cystectomy.
• Comparing cancer-specific survival and overall survival in RCTs in patients receiving trimodal therapy vs. radical cystectomy.
• Comparing clinical T stage and Eastern Cooperative Oncology Group (ECOG) in patients receiving trimodal therapy vs. radical cystectomy, T stage <2 for RCT, (B) T stage <2 for retrospective studies, T stage ≥2 for RCT, T stage ≥2 for retrospective studies, ECOG ≥1, and ECOG = 0 in patients receiving trimodal therapy vs. radical cystectomy.
